# A Mechanistic Study of the Osteogenic Effect of Arecoline in an Osteoporosis Model: Inhibition of Iron Overload-Induced Osteogenesis by Promoting Heme Oxygenase-1 Expression

**DOI:** 10.3390/antiox13040430

**Published:** 2024-03-31

**Authors:** Zhongjing Jiang, Linhua Deng, Gang Xiang, Xia Xu, Yunjia Wang

**Affiliations:** 1 Department of Spine Surgery and Orthopaedics, Xiangya Hospital, Central South University, Changsha 410008, China; zhongjing@csu.edu.cn (Z.J.); 228111085@csu.edu.cn (L.D.); csuxyxg@csu.edu.cn (G.X.); 2 National Clinical Research Center for Geriatric Disorders, Xiangya Hospital, Central South University, Changsha 410008, China; 3 Department of General Practice, Xiangya Hospital, Central South University, Changsha 410008, China

**Keywords:** osteoporosis, iron overload, ferroptosis, arecoline, heme oxygenase-1

## Abstract

Iron overload-associated osteoporosis presents a significant challenge to bone health. This study examines the effects of arecoline (ACL), an alkaloid found in areca nut, on bone metabolism under iron overload conditions induced by ferric ammonium citrate (FAC) treatment. The results indicate that ACL mitigates the FAC-induced inhibition of osteogenesis in zebrafish larvae, as demonstrated by increased skeletal mineralization and upregulation of osteogenic genes. ACL attenuates FAC-mediated suppression of osteoblast differentiation and mineralization in MC3T3-E1 cells. RNA sequencing analysis suggests that the protective effects of ACL are related to the regulation of ferroptosis. We demonstrate that ACL inhibits ferroptosis, including oxidative stress, lipid peroxidation, mitochondrial damage, and cell death under FAC exposure. In this study, we have identified heme oxygenase-1 (HO-1) as a critical mediator of ACL inhibiting ferroptosis and promoting osteogenesis, which was validated by HO-1 knockdown and knockout experiments. The study links ACL to HO-1 activation and ferroptosis regulation in the context of bone metabolism. These findings provide new insights into the mechanisms underlying the modulation of osteogenesis by ACL. Targeting the HO-1/ferroptosis axis is a promising therapeutic approach for treating iron overload-induced bone diseases.

## 1. Introduction

Ferric ammonium citrate-facilitated iron overload (FAC) is a well-established experimental model for the study of the pathophysiology of iron overload disorders [1,2], including osteoporosis [3]. Osteoporosis is a prevalent bone disorder that affects millions of people worldwide, particularly postmenopausal women and the elderly. It is characterized by an imbalance between bone formation and resorption, resulting in decreased bone density and an increased risk of fracture [4]. This disrupts bone homeostasis and creates an unfavorable microenvironment for bone tissue, contributing to the development of osteoporosis. Excessive iron deposition can impair the function of osteoblasts and stimulate the activity of osteoclasts, which ultimately leads to a decrease in bone formation and an increase in bone resorption [5,6].

Recent studies have shown an increased interest in the role of arecoline (ACL), a natural alkaloid found in the areca nut, in the study of bone metabolism [7,8,9]. Although chewing areca nut is a common habit in many regions, it has been associated with adverse health effects, including oral cancer and cardiovascular disease [10]. However, the direct impact of ACL on bone metabolism is still not well understood [11]. Arecoline has been shown to suppress RANKL-induced osteoclast differentiation in vitro and attenuate LPS-induced bone loss in vivo [7], suggesting that it may have beneficial effects on bone health.

Ferroptosis is a form of cell death that is characterized by iron-dependent lipid peroxidation and oxidative stress. It has recently been identified as a significant factor in bone metabolism research [12]. The association between ferroptosis and osteoporosis lies in the dysregulation of iron metabolism within osteoblasts and osteoclasts [13]. In osteoporotic conditions, disturbances in iron homeostasis can tip the balance toward ferroptosis. This can exacerbate bone loss and impair bone formation. The interaction between ferroptosis and osteoporosis creates a damaging cycle, as bone loss exacerbates oxidative stress and ferroptosis, perpetuating bone deterioration [14].

The aim of this study was to investigate the effects of ACL on bone metabolism and ferroptosis in the presence of FAC-induced iron overload, and to elucidate the molecular mechanisms underlying the protective effects of ACL and its potential to counteract iron overload-associated osteoporosis. We used FAC-treated zebrafish larvae as an in vivo model to simulate iron overload-induced osteoporosis [15]. MC3T3-E1 cells were used to study the molecular pathways of ACL’s promotion of osteogenic differentiation under iron overload conditions. In addition, we used RNA-seq analysis to explore potential pathways involved in the anti-osteoporosis effects of ACL under FAC exposure. Our results suggest that ACL inhibits ferroptosis and benefits bone metabolism under iron overload, possibly via upregulation of HO-1.

## 2. Methods and Materials

### 2.1. Materials and Reagents

FAC (E0375), Arecoline (S2614), Fer-1 (S7243), NAC (S1623), and GSH (S4606) were purchased from Selleck (Shanghai, China). Calcein was purchased from Dojin (Tokyo, Japan). The Cell Counting Kit-8 (CCK-8) assay kit was also purchased from Dojin (Japan). Maleic dialdehyde (MDA), DCFH-DA assay kits, Alizarin red S, alkaline phosphatase (ALP), and diethanolamine activity kit were obtained from Sigma-Aldrich (St. Louis, MO, USA). Antibodies against HO-1 (ab189491), GPX4 (ab125066), SLC7A11 (ab175186), and β-actin (ab8226) were purchased from Abcam (Cambridge, MA, USA). MC3T3-E1 cells (Procell Life Sci.&Tech. Co., Ltd., Wuhan, China) were incubated in MEM with 10% FBS, 10 mM glutamine, and 100 U/mL penicillin/streptomycin. For osteogenic differentiation, the culture medium was replaced with MEM containing 10% FBS, 10 mM β-glycerol phosphate, 50 μM ascorbic acid, 100 nM dexamethasone, and 100 U/mL penicillin/streptomycin.

### 2.2. Zebrafish Husbandry, Treatment and Locomotion Analysis

The Institutional Animal Care and Use Committee (IACUC) at Xiangya Hospital, Central South University reviewed and approved all in vivo procedures and protocols. Zebrafish (*Danio rerio*) were bred and maintained in a controlled environment with a 14/10 h light-dark cycle at a constant temperature of 28 °C in a flow-through system. Standard fertilization protocols were followed to obtain zebrafish embryos. At 1 dpf, zebrafish embryos were randomly assigned to six groups and exposed to ACL concentrations ranging from 0.01 to 10 mg/L. The ACL solutions were renewed every 24 h until 5 or 10 dpf to ensure continuous exposure. We recorded survival, hatching, and malformation rates of the zebrafish larvae. At 5 dpf, zebrafish embryos were randomly divided into different treatment groups and treated with ACL at specified concentrations (0.01–10 mg/L) for 24 h. A video tracking system was used to perform locomotion analysis on zebrafish larvae. The analysis recorded and analyzed swimming trajectories, average speed, and total movement distance. Zebrafish and embryo specimens from each treatment group were imaged using a Motic SM7 microscope (Motic, Xiamen, China).

### 2.3. Generation of the Transgenic Zebrafish Line

To create the *hmox1a*^−/−^ zebrafish line, a gene knockout approach was employed using CRISPR/Cas9 technology. Briefly, Guide RNAs targeting the *hmox1a* gene were designed (see Supplementary Table S1 for guide RNA sequences), and Cas9 protein was injected into zebrafish embryos at the one-cell stage. The injected embryos were raised to adulthood, and mutations in the *hmox1a* gene were screened for in F0 founders. F0 founders with targeted mutations were crossed with wildtype zebrafish to generate F1 progeny. The F1 progeny were genotyped, and homozygous *hmox1a*^−/−^ mutants were selected as the transgenic zebrafish line for subsequent experiments.

### 2.4. Calcein Staining

Zebrafish larvae (7 dpf) from various treatment groups were exposed to a 20 µM calcein solution in embryo medium for 24 h in a dark environment for 10 min. The larvae were then washed with fresh embryo medium to remove any excess calcein and imaged using a fluorescence microscope with consistent exposure settings. The fluorescence intensities of vertebral bodies were quantified using image analysis software(Image J version 1.53c).

### 2.5. qPCR Analysis

Quantitative real-time polymerase chain reaction (qPCR) was conducted to evaluate the mRNA expression levels of key osteogenic markers (Runx2, osteocalcin, osteopontin), ferroptosis-related genes (*slc34a2a*, *loc558816*, *acsl4a*), apoptosis-related genes (*p53*, *casp3a and casp9*) and ho-1(*hmox1a* in zebrafish) in zebrafish larvae and MC3T3-E1 cells. Total RNA was isolated from the samples using a commercial RNA extraction kit, and cDNA was synthesized using a reverse transcription kit. qPCR was performed with specific primers for the target genes (Primers detail see Supplementary Table S2), and the relative gene expression levels were calculated using the 2^−ΔΔCt^ method.

### 2.6. Cell Proliferation Assay

The impact of ACL on cell proliferation was assessed using the Cell Counting Kit-8 (CCK-8) assay. MC3T3-E1 cells were exposed to varying concentrations of FAC, ACL, or Fer1 for 72 h. The CCK-8 reagent was then added to the cells, and the absorbance was measured at a specific wavelength using a microplate reader. Cell viability was calculated based on the absorbance values, indicating the effect of ACL and Fer1 on cell proliferation.

### 2.7. Alizarin Red and Alkaline Phosphatase Staining

MC3T3-E1 cells were cultivation according to standard protocols. To assess osteogenic differentiation, fixed cells were stained with Alizarin red and alkaline phosphatase (ALP) following the manufacturer’s protocols. For ALP, cells were stained for 20 min and then ALP activity was quantified using a kit after 10 days of cultivation. For Alizarin red, cells were stained for 15 min and imaged after 20 days of cultivation. To quantify calcium deposits, treated cells were incubated with 10% cetylpyridinium chloride (CPC) for 2 h at 37 °C. After incubation, the CPC solution was transferred to a 96-well plate and the absorbance was measured at OD = 570 nm using a plate reader (BD Biosciences, San Jose, CA, USA).

### 2.8. RNA Sequencing and Data Analysis

Zebrafish larvae (5 dpf) were exposed to 200 μg/mL of ferric ammonium citrate (FAC) with or without 0.5 mg/L of arecoline. After 48 h of exposure, total RNA was extracted from thirty larvae using Trizol (Takara Biochemicals, Dalian, China). The mRNA was enriched, fragmented, and reverse transcribed into cDNA. The cDNA was then modified with adapters and amplified via PCR. The double-stranded PCR products were denatured by heat and then circularized using the splint oligo sequence, resulting in the final library. The cDNA library was sequenced on the BGISEQ-500 platform at BGI tech (BGI-Shenzheng, China). The raw sequencing data was processed and filtered using SOAPnuke (v1.5.2) to obtain clean reads in FASTQ format. The reads were mapped to the reference genome using HISAT2 (v2.0.4), and RSEM (v1.2.12) was used to quantify the expression level of each gene. A thorough analysis of the sequencing data was conducted, including enrichment analysis of differentially expressed genes using Gene Ontology (GO) (US National Institutes of Health, US) and Kyoto Encyclopedia of Genes and Genomes (KEGG) (Kanehisa Laboratories, Japan). The significance levels of enriched terms and pathways were corrected using a Q value threshold to ensure rigorous statistical analysis.

### 2.9. ROS Production Measurement

To assess ROS production in zebrafish embryos, we used the fluorescent dye 2′,7′-dichlorofluorescein diacetate (DCF-DA). Embryos from different treatment groups were incubated with 10 µM DCF-DA in embryo medium for 30 min in the dark at 5 dpf. Afterward, embryos were washed with fresh embryo medium and imaged using a fluorescence microscope. We quantified fluorescence intensity using image analysis software.

### 2.10. Quantification of Iron, Malondialdehyde (MDA), and Glutathione (GSH) Levels in Zebrafish Larvae

To elucidate the effects of ACL on oxidative stress and iron homeostasis in zebrafish larvae under FAC exposure, we performed quantification of intracellular iron, MDA, and GSH levels. We conducted specific biochemical assays on zebrafish larvae treated with different drug combinations. Iron levels were measured using colorimetric assays, while MDA levels were determined using the thiobarbituric acid reactive substances (TBARS) assay. Additionally, we used a commercially available kit to determine GSH levels.

### 2.11. Transmission Electron Microscopy (TEM)

MC3T3-E1 cells underwent various intervention techniques to ensure viability before being transferred to 6-well plates for 72 h to investigate possible cellular ultrastructural changes induced by ACL and FAC treatments. For transmission electron microscopy (TEM) analysis, ultrathin sections (50–70 nm thick) were carefully prepared from the treated cells and sequentially stained with 2% uranyl acetate and lead citrate. The purpose of this action was to improve contrast and aid in the visualization of cellular structures. The ultrastructure of the cell was observed and imaged using a high-resolution transmission electron microscope (FEI F200C, Hillsboro, OR, USA).

### 2.12. Oxidative Stress Analysis

Specific assays were used to assess oxidative stress in zebrafish embryos and MC3T3-E1 cells. Fluorescent probes, DCF-DA, were used to determine levels of reactive oxygen species (ROS). These probes fluoresce upon reaction with ROS. The fluorescence intensity was quantified using a fluorescence microscope or a microplate reader to determine ROS levels in the samples.

### 2.13. Intracellular ROS, Iron, Lipid Peroxidation Levels Determination

Intracellular levels of reactive oxygen species (ROS), iron, and lipid peroxidation were assessed using specific fluorescent probes or commercial assay kits. To detect ROS, cells were incubated with fluorescent probes such as DCF-DA or DHE (dihydroethidium), and fluorescence intensity was measured using a fluorescence microscope or microplate reader. Iron and lipid peroxidation levels were measured using commercially available assay kits. The levels of absorbance or fluorescence intensity were quantified to determine their respective values in the samples.

### 2.14. siRNA Transfection

To achieve targeted knockdown of heme oxygenase-1 (HO-1) in MC3T3-E1 cells, we used small interfering RNA (siRNA) specific to HO-1. The siRNA was transfected into the cells using Lipofectamine 2000, resulting in knockdown of HO-1 gene expression. MC3T3-E1 cells were transfected with HO-1-siRNA (GenePharma, Shanghai, China) (Supplementary Table S3) for 24 h before undergoing the FAC + ACL treatment.

### 2.15. Western Blot Analysis

Quantification of protein expression pertaining to HO-1 and genes associated with ferroptosis (GPX4 and SLC7A11) was conducted through Western blotting. RIPA buffer, fortified with protease inhibitors, was employed for lysing MC3T3-E1 cells. Subsequent to separation via SDS-PAGE (10 μL of sample was loaded per well, and protein concentrations were adjusted to 1 mg/mL), the protein lysates were translocated onto PVDF membranes. These membranes underwent incubation with primary antibodies targeting HO-1 (1:1000), GPX4 (1:5000), SLC7A11 (1:10,000), or β-actin (loading control) at 1:10,000 dilutions. Following thorough washing, secondary antibodies, conjugated with horseradish peroxidase, were applied. The visualization of protein bands was accomplished through enhanced chemiluminescence, with band intensities quantified using image analysis software.

### 2.16. Statistical Analysis

All data were presented as mean ± standard deviation (SD) from at least three independent experiments. Statistical analysis was performed using GraphPad Prism software version 10.1.2. One-way analysis of variance (ANOVA) followed by Tukey’s post hoc test was used for multiple group comparisons. Differences were considered statistically significant at *p* < 0.05.

## 3. Results

### 3.1. ACL Exposure Caused Concentration-Dependent Developmental Malformations in Zebrafish

**Figure 1:**
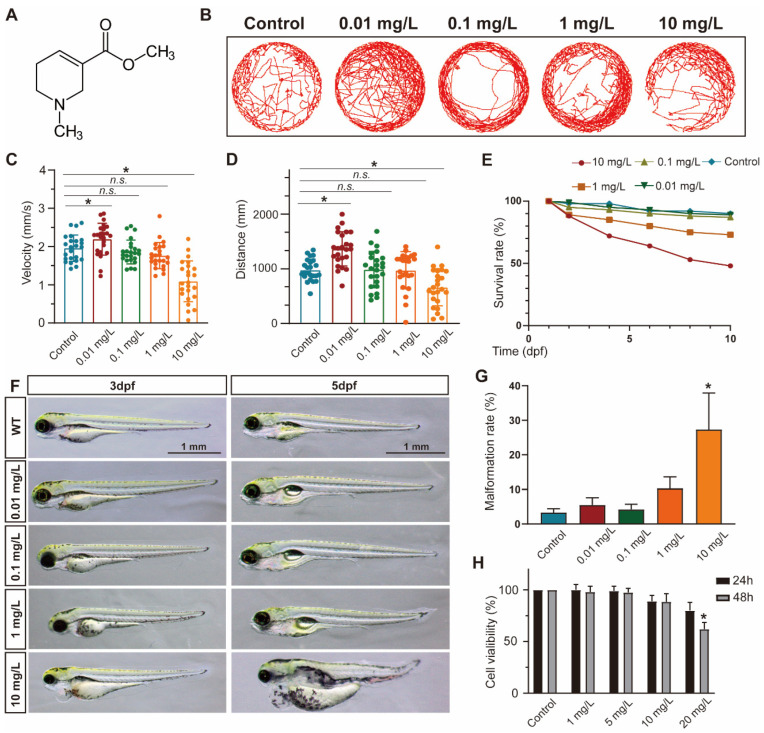
ACL exposure caused developmental malformations in zebrafish at high concentrations. (**A**) The chemical structure of ACL. (**B**) Locomotion tracks, (**C**) average speed and (**D**) total movement distance of zebrafish larvae exposed to 0.01–10 mg/L ACL treatment at 5 dpf (*n* = 25). (**E**) Zebrafish embryos survival proportions treated with 0.01–10 mg/L ACL for 10 days. (**F**) Malformation phenotypes of embryos exposed to 0.01–10 mg/L ACL for 3–5 days. (**G**) Malformation rate of different concentrations of ACL-treated embryos in 5 dpf (*n* = 50). (**H**) Cell viability of MC3T3-E1 cells under various concentrations of ACL for 24 h or 48 h, blank was treated as the control group. Data are expressed as the mean ± standard deviation (SD), * *p* < 0.05 versus the control group.

To evaluate the impact of ACL exposure on zebrafish activity, we recorded the locomotor tracks of zebrafish larvae exposed to different concentrations of ACL (0.01–10 mg/L) at 5 days post-fertilization (dpf) and analyzed their average speed and total movement distance (Figure 1B–D). Exposure to ACL resulted in changes to locomotor patterns, including a decrease in average speed and total distance traveled. The effects were concentration dependent. Additionally, we determined the survival rates of zebrafish embryos treated with various concentrations of ACL for 10 days. The results indicated a concentration-dependent decrease in survival rates with ACL concentrations above 1 mg/L, with a survival rate of only 48% observed when treated with 10 mg/L ACL for 10 days (Figure 1E). Exposure to high concentrations of ACL resulted in malformation phenotypes in zebrafish embryos. Malformations such as pericardial edema, typical of zebrafish toxicity, were detected (Figure 1F). The malformation rate was analyzed in embryos exposed to different ACL concentrations for 3–5 days. The malformation rate increased significantly when the ACL concentration was higher than 1 mg/L, reaching 27.3% when treated with 10 mg/L ACL for 5 days (Figure 1G). The results suggest that ACL concentrations higher than 1 mg/L have a biological effect on zebrafish. To assess the cytotoxicity of ACL on mammalian cells, we evaluated the cell viability of MC3T3-E1 cells under different ACL concentrations for 24 and 48 h. Treatment with 20 mg/mL ACL for 48 h resulted in a significant decrease in cell activity (62.0 ± 6.6%) (Figure 1H).

### 3.2. ACL Alleviated FAC-Induced Inhibition of Osteogenesis in Zebrafish

**Figure 2:**
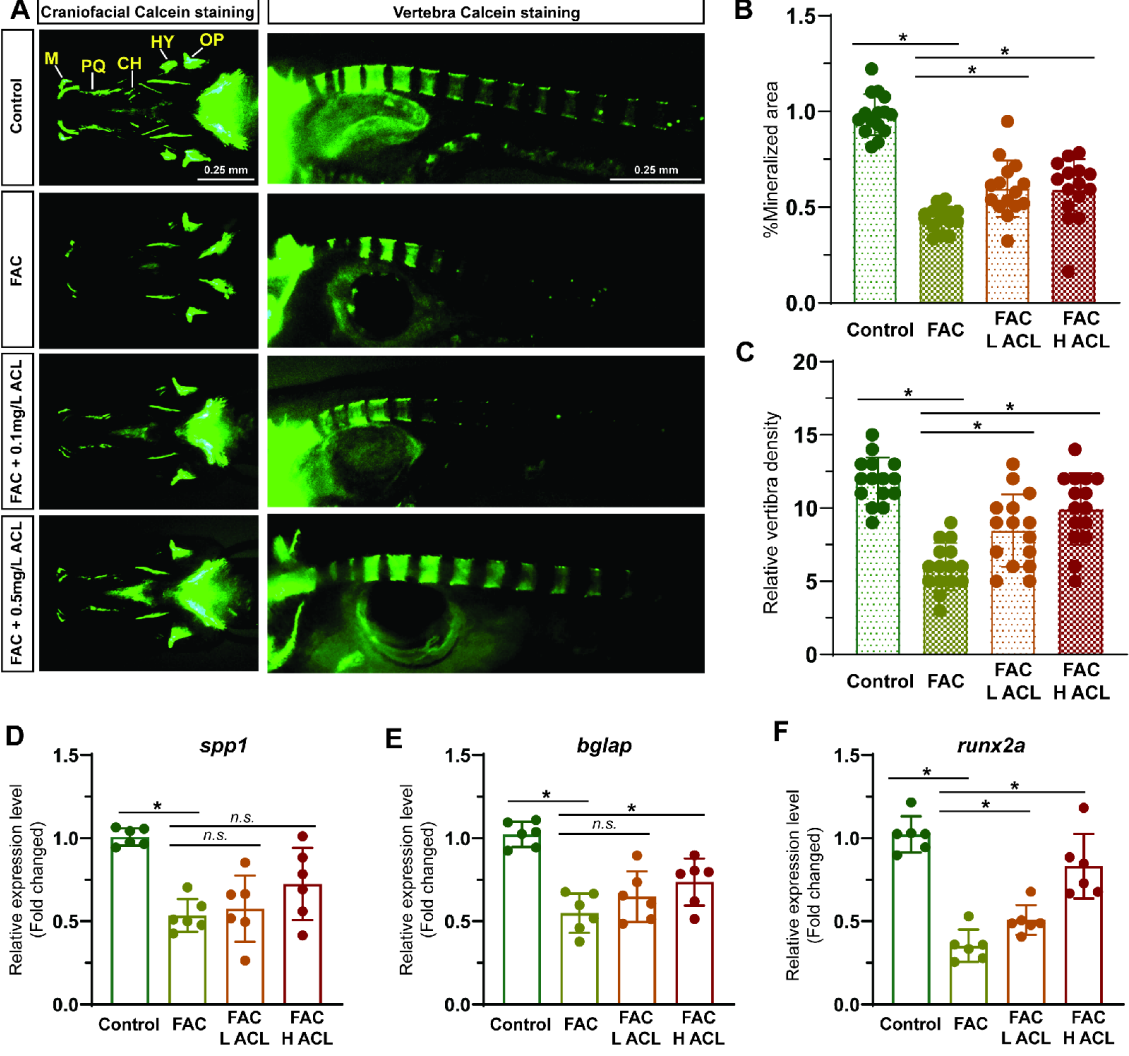
ACL attenuated the inhibition effect of FAC in bone formation in zebrafish. (**A**) Calcein staining of zebrafish larvae (7 dpf) cranium from control, FAC (200 µg/mL), FAC with low (0.1 mg/L) or high (0.5 mg/L) concentration ACL treatment groups. (**B**) Mineralized areas were quantified and shown in the graph. (**C**) The number of calcein-stained vertebra bodies were calculated. (**D**–**F**) The relative mRNA expression of spp1, bglap and runx2a in zebrafish larvae treated with blank medium, FAC, FAC + high (0.5 mg/L) or low (0.1 mg/L) ACL were determined by qPCR. The mean value of blank medium-only treated group was set to 1.0. Data are showed as mean ± standard error. * means *p* < 0.05 between two indicated groups (*n* = 6). M, Meckel; PQ, palatoquadrate; CH, ceratohy; HY, hyomandibula (hyosympletic); OP, opercula.

Alizarin red staining of zebrafish larvae cranium at 7 dpf revealed that exposure to FAC resulted in decreased mineralization (Figures S1 and S2). However, co-treatment with low (0.1 mg/L) or high (0.5 mg/L) concentrations of ACL attenuated this inhibitory effect (Figure 2A). The quantification results confirmed that mineralized areas increased by 46%, and calcein-stained vertebral bodies increased from 6.0 ± 1.6 to 9.9 ± 2.5 after ACL (0.5 mg/L) treatment (Figure 2B,C). To validate the osteogenic effect of ACL, we determined the relative mRNA expression of key osteogenic markers spp1, bglap, and runx2a. Treatment with (0.5 mg/L) ACL upregulated bglap and runx2a, indicating the promotion of osteogenesis (Figure 2D–F). These results demonstrate that ACL treatment effectively attenuated the inhibitory effects of FAC on osteogenesis in zebrafish larvae.

### 3.3. ACL Improved the Osteogenic Differentiation of MC3T3-E1 Cells under FAC Treatment

**Figure 3:**
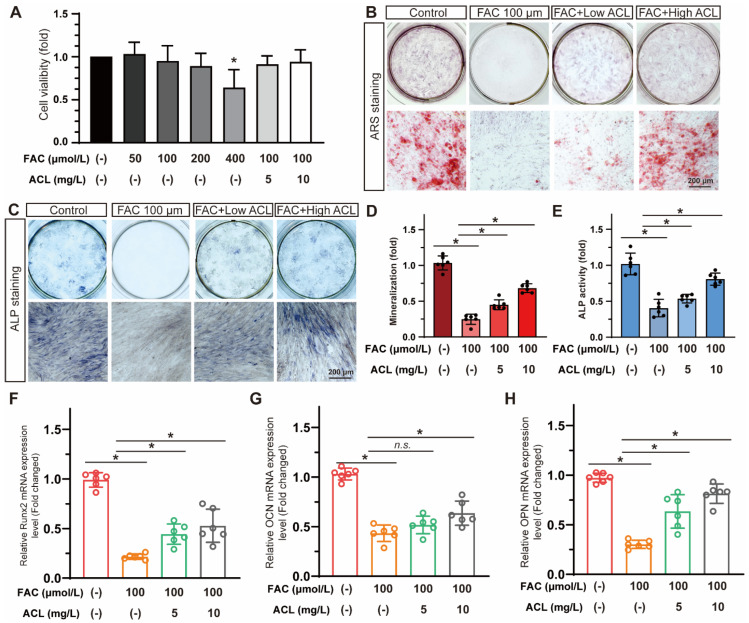
ACL promoted the osteogenic differentiation of MC3T3-E1 cells under FAC treatment. (**A**) Cell viability of MC3T3-E1 cells treated with different concentrations of FAC (0–400 µmol/L) with or without ACL (5 or 10 mg/L) for 24 h. * *p* < 0.05 versus control group. (**B**,**C**) Alizarin red staining and Alkaline phosphatase staining of MC3T3-E1 cells treated with 100 μmol/L FAC and exposed to 5 or 10 mg/L ACL for 1 or 2 weeks. (**D**,**E**) Degree of mineralization and Alkaline phosphatase activity were quantified, * means *p* < 0.05 between two indicated groups (*n* = 6). (**F**–**H**) Quantitative analysis of mRNA expression levels of runt-related transcription factor 2 (Runx2), osteocalcin (OCN) and osteopontin (OPN) in MC3T3-E1 cells cultured in 100 μmol/L FAC with 5 or 10 mg/L ACL for 3 days. The control group was set to 1.0, * means *p* < 0.05 between two indicated groups (*n* = 6). Data are displayed as mean ± SD.

Cell viability was assessed in MC3T3-E1 cells exposed to varying concentrations of FAC (0–400 µmol/L) with or without ACL (5 or 10 mg/L) for 24 h. Treatment with less than 200 mg/L FAC and 10 mg/L ACL was found to be safe for MC3T3-E1 cells (Figure S2). However, treatment with 400 µmol/L FAC decreased cell viability to 64 ± 21% (Figure 3A). The study evaluated the effects of ACL on osteogenic differentiation by conducting alizarin red staining and alkaline phosphatase staining of MC3T3-E1 cells treated with FAC and ACL. The results showed that ACL-treated cells exhibited enhanced mineralization and increased alkaline phosphatase activity compared to the FAC-only treated group (Figure S1). Specifically, mineralization increased 2.62-fold and ALP activity increased 1.88-fold (Figure 3B–E). To investigate the molecular mechanism underlying the osteogenic effects of ACL, we analyzed the mRNA expression levels of runt-related transcription factor 2 (Runx2), osteocalcin (OCN), and osteopontin (OPN) in MC3T3-E1 cells cultured in 100 μmol/L FAC with 5 or 10 mg/L ACL for 3 days. Our results showed that treatment with ACL (10 mg/L) significantly upregulated the expression of Runx2, OCN, and OPN under FAC exposure, suggesting an enhancement in osteogenic gene expression (Figure 3F–H). Overall, the results indicate that ACL enhances osteogenic differentiation of MC3T3-E1 cells under FAC treatment.

### 3.4. Ferroptosis May Play a Role in the Anti-Osteoporotic Effects of ACL under FAC Exposure

**Figure 4:**
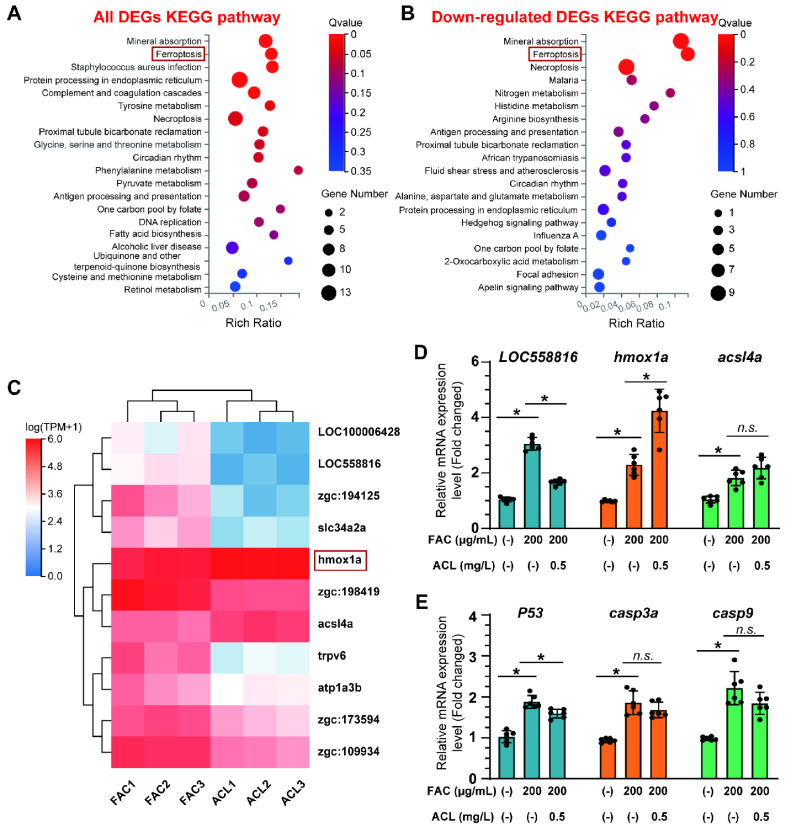
RNA-seq identified that ferroptosis may be involved in anti-osteoporosis effect of ACL under FAC exposure. (**A**) KEGG pathway enrichment analysis of all differentially expressed genes (DEGs) between ACL and ACL + FAC groups. (**B**) KEGG pathway enrichment analysis of down-regulated genes in ACL + FAC treated zebrafish larvae compared to ACL treated larvae. (**C**) Heatmap of 11 ferroptosis-related genes identified from DEGs. FAC represents the group treated with FAC alone, while ACL represents the group treated with both FAC and ACL. Each row represents one gene, and each column represents one sample. Red represents genes with high expression, blue represents genes with low expression. Darker colors indicate more significant differences. (**D**) Verification of mRNA expression level of ferroptosis-related genes in 5 dpf zebrafish larvae treated with 200 µg/mL FAC with or without 0.5 mg/L ACL. (**E**) Quantitative analysis of mRNA expression levels of three apoptosis genes, *P53*, *casp3a* and *casp9*. * means *p* < 0.05 between two indicated groups (*n* = 6). Data are displayed as mean ± SD.

To gain a deeper understanding of the molecular mechanisms underlying the osteoprotective effects of ACL, we conducted RNA sequencing in zebrafish larvae with distinct osteogenic responses under FAC exposure with or without ACL. The sequencing analysis revealed an enrichment of several pathways related to osteogenesis, particularly mineral uptake, suggesting a potential mechanism by which ACL promotes bone formation (Figure 4A). Among the downregulated pathways, a more pronounced difference was observed in the ferroptosis pathway, suggesting a potential link between ACL treatment and iron metabolism (Figure 4B). To identify key genes involved in the effects of ACL, we focused on the expression patterns of ferroptosis-related genes. The expression of *hmox1a*, a critical regulator of iron homeostasis, was notably upregulated after ACL treatment (Figure 4C). To clarify the association between ferroptosis and ACL treatment, we analyzed the expression of three ferroptosis-related genes and three apoptosis-related genes. The results revealed that ACL treatment led to a significant increase in *hmox1a* expression and decrease in *loc558816* expression, suggesting a potential involvement of these genes in mediating the effects of ACL. However, there was no statistically significant difference in the expression of the ferroptosis-related gene *acsl4a* (Figure 4D). With regard to apoptosis-related genes, ACL treatment resulted in only a slight decrease in *p53* expression, indicating a potential minor impact on apoptosis (Figure 4E). Thus, our results suggest that the primary effect of ACL may be on ferroptosis rather than apoptosis.

### 3.5. Ferroptosis Was Inhibited by ACL Treatment under FAC Exposure

**Figure 5:**
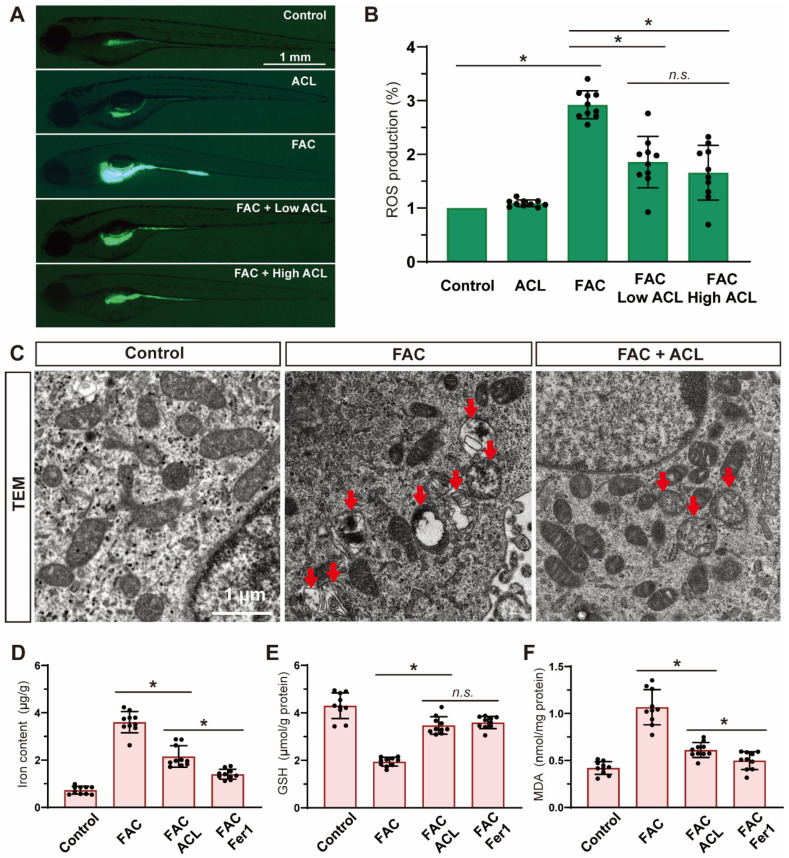
Ferroptosis was inhibited by ACL treatment under FAC exposure. (**A**,**B**) ROS production of zebrafish embryos (5 dpf) from control, FAC and FAC + (0.25 or 0.5 mg/L) ACL groups was detected and quantified by staining with DCF-DA, * means *p* < 0.05 between two indicated groups (*n* = 10). (**C**) Mitochondria ultrastructure of zebrafish embryo sections from control, FAC and FAC + ACL groups was imaged by transmission electron microscopy. Red arrows indicate damaged mitochondrial, including mitochondrial swelling, cristae lysis, and ruptured outer membrane. (**D**–**F**) Iron, GSH and MDA levels were determined in whole zebrafish embryos (5 dpf) from control, FAC, FAC + ACL and FAC + Fer1 groups, the concentrations used in the experiments were as follows: FAC (200 μg/mL), ACL (0.5 mg/L), and Fer1 (1 μM), data were displayed as mean ± SD, * means *p* < 0.05 between two indicated groups (*n* = 10).

To evaluate the potential inhibitory effects of ACL on reactive oxygen species (ROS) production induced by FAC, ROS levels were measured in zebrafish embryos at 5 dpf from different treatment groups using DCF-DA staining. The level of ROS significantly decreased (reduced to 55%) after treatment with 0.5 mg/L ACL (Figure 5A,B and Figure S5), indicating the effectiveness of ACL in suppressing ROS generation induced by FAC exposure. Additionally, we used transmission electron microscopy to evaluate the ultrastructure of mitochondria in zebrafish embryo sections to gain further insights into ACL’s impact on ferroptosis. The group treated with ACL displayed intact mitochondrial ultrastructure, while the group treated with FAC exhibited signs of mitochondrial damage consistent with ferroptosis, including mitochondrial swelling, cristae lysis, and ruptured outer membrane (Figure 5C). Iron, glutathione (GSH), and malondialdehyde (MDA) levels were quantified in whole zebrafish embryos at 5 dpf. ACL treatment significantly decreased iron (3.72 ± 0.45 vs. 2.23 ± 0.31 μg/g) and MDA (1.11 ± 0.18 vs. 0.47 ± 0.10 μg/g) levels, while concurrently elevating GSH levels (2.05 ± 0.16 vs. 3.63 ± 0.51 μmol/g), when compared to the FAC group (Figure 5D–F). These findings support the role of ACL as an inhibitor of ferroptosis under FAC exposure. The reduction in iron levels indicates ACL’s ability to counteract iron-induced lipid peroxidation, ultimately attenuating oxidative stress associated with ferroptosis. Overall, our results demonstrate that ACL treatment effectively inhibits ferroptosis in zebrafish embryos exposed to FAC.

### 3.6. Inhibition of Ferroptosis by ACL Can Be Reversed by HO-1 Inhibition in MC3T3-E1 Cells

**Figure 6:**
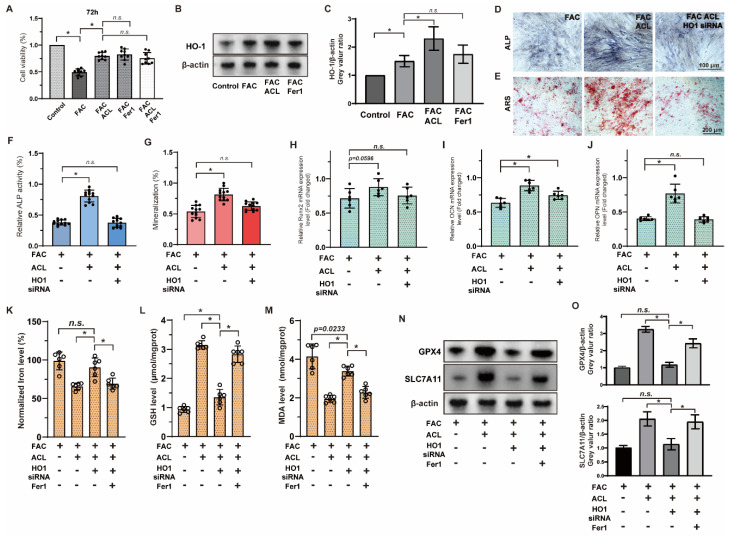
Inhibition of ferroptosis by ACL can be reversed by HO-1 inhibition. (**A**) The survival of MC3T3-E1 cells were examined using the Cell Counting Kit-8 assay. Cells were treated with FAC, FAC + ACL, FAC + Fer1 (Ferroptosis inhibitor) or FAC + ACL + Fer1 for 72 h. (**B**,**C**) The expression of HO-1 in control, FAC, FAC + ACL and FAC + Fer1 groups were analyzed by Western blotting. β-actin was used as internal reference. Relative expression levels were shown in the graph. * means *p* < 0.05 between two indicated groups (*n* = 3). (**D**,**E**) Alizarin red staining and Alkaline phosphatase staining of MC3T3-E1 cells treated with FAC, FAC + ACL or FAC + ACL + HO-1 siRNA (HO-1 knockdown) for 1 or 2 weeks. (**F**,**G**) Degree of mineralization and Alkaline phosphatase activity were quantified, * means *p* < 0.05 between two indicated groups (*n* = 6). (**H**–**J**) Quantitative analysis of mRNA expression levels of runt-related transcription factor 2 (Runx2), osteocalcin (OCN) and osteopontin (OPN) in MC3T3-E1 cells cultured in FAC (100 μmol/L), FAC + ACL (10 mg/L) or FAC + ACL + HO-1 siRNA (HO-1 knockdown) for 3 days. The control group was set to 1.0, * means *p* < 0.05 between two indicated groups (*n* = 6). (**K**–**M**) Intracellular iron, GSH (Glutathione), lipid peroxidation MDA (Malondialdehyde) levels in FAC, FAC + ACL, FAC + ACL + HO-1 siRNA and FAC + ACL + HO-1 siRNA + Fer1 treated MC3T3-E1 cells were detected by commercialized assay kits. * means *p* < 0.05 between two indicated groups (*n* = 6). (**N**,**O**) The expression of ferroptosis associated genes, GPX4 and SLC7A11, in FAC, FAC + ACL, FAC + ACL + HO-1 siRNA and FAC + ACL + HO-1 siRNA + Fer1 treated MC3T3-E1 cells were determined by Western blotting. β-actin was used as internal reference. Relative expression levels were shown in the graph. The concentrations used in the experiments were as follows: FAC (100 μM), ACL (0.5 mg/L), and Fer1 (1 μM) * means *p* < 0.05 between two indicated groups (*n* = 3). All data are displayed as mean ± SD.

To evaluate the impact of ACL on cell survival and its potential role in inhibiting ferroptosis, we conducted a Cell Counting Kit-8 assay in MC3T3-E1 cells to compare the effects of ACL (0.25 or 0.5 mg/L) and Fer1 (1 μM) on cell activity under FAC (100 μM). Remarkably, ACL treatment significantly promoted cell survival (from 49.03 ± 6.56 to 79.69 ± 6.73%), an effect that mirrored the treatment with Fer1. We did not observe any synergistic interaction between ACL and Fer1, indicating that ferroptosis inhibition is involved in ACL’s protective mechanism (Figure 6A). Sequencing results suggested that HO-1 (heme oxygenase-1) may be involved in ACL-mediated regulation of ferroptosis. To further explore this, we analyzed HO-1 expression under FAC and ACL treatment in different groups (control, FAC, FAC + ACL, and FAC + Fer1) using Western blotting. HO-1 expression was upregulated 1.51-fold by ACL treatment compared to FAC treatment. However, Fer1 group did not show any effect on HO-1 expression, indicating that the inhibition of ferroptosis does not directly impact HO-1 expression. To gain more insight into the significance of HO-1 in ACL’s protective effect, we performed Alizarin red and alkaline phosphatase staining on MC3T3-E1 cells with either normal HO-1 expression or HO-1 knockdown, treated with FAC with or without ACL for 1 or 2 weeks. Our results revealed that HO-1 knockdown partially inhibited the promotional effect of ACL on mineralization and alkaline phosphatase activity (Figure 6D–G and Figure S3). Additionally, we conducted a quantitative analysis of mRNA expression levels of key osteogenic markers, namely Runx2, OCN, and OPN. The results showed that HO-1 knockdown attenuated the upregulation of these markers induced by ACL treatment (Figure 6H–J). To further validate the regulation relationship between ACL and ferroptosis, we measured intracellular iron, GSH, and MDA levels in normal or HO-1 knockdown MC3T3-E1 cells treated with FAC with or without ACL. HO-1 knockdown partially inhibited the alterations in ferroptosis-associated factors induced by ACL treatment (Figure 6K–M). Additionally, Western blot analysis of ferroptosis-associated genes, GPX4 and SLC7A11, revealed that HO-1 knockdown restored the downregulation of these genes induced by ACL treatment. This effect was partially reversed by co-treatment with Fer1 (Figure 6N,O and Figure S4). These observations suggest that ACL regulates ferroptosis via HO-1 overexpression.

### 3.7. The Protective Effect of ACL Can Be Inhibited by hmox1a Knockout in Zebrafish

**Figure 7:**
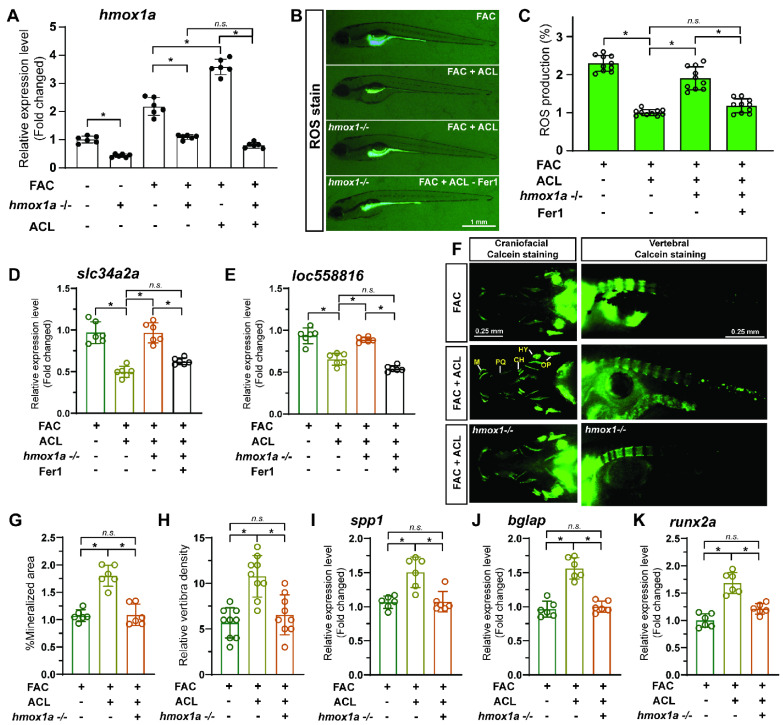
The protective effect of ACL can be inhibited by *hmox1a* knockout in zebrafish. (**A**) Zebrafish hmox1a mRNA level in control, FAC, FAC + ACL treated wildtype or *hmox1a*^−/−^ larvae were analyzed by qPCR, * *p* < 0.05. (**B**,**C**) ROS production of embryos (5dpf) from two zebrafish lines, wildtype and *hmox1a*^−/−^ larvae treated with FAC, FAC + ACL and FAC + ACL + Fer1 were detected and quantified by staining with DCF-DA, * means *p* < 0.05 between two indicated groups (*n* = 10). (**D**,**E**) Determination of mRNA expression level of ferroptosis-related genes in 5 dpf zebrafish larvae or *hmox1a*^−/−^ larvae treated with 200 µg/mL FAC with or without 0.5 mg/L ACL. (**F**) Calcein staining images of wildtype and *hmox1a*^−/−^ zebrafish larvae (7 dpf) treated with FAC, FAC + ACL. (**G**,**H**) The fluorescence intensity of vertebrae of zebrafish larvae in different groups were quantified and compared. * means *p* < 0.05 between two indicated groups (*n* = 15). (**I**–**K**) The relative mRNA expression of zebrafish osteogenesis related genes, spp1, bglap and runx2a, in wildtype and *hmox1a*^−/−^ zebrafish larvae treated with FAC, FAC + ACL were determined by qPCR. The mean value of FAC treated wildtype group was set to 1.0. The concentrations used in the experiments were as follows: FAC (200 μg/mL), ACL (0.5 mg/L), and Fer1 (1 μM), * means *p* < 0.05 between two indicated groups (*n* = 6). Data are displayed as mean ± SD.

To explore the crucial role of *hmox1a* in the protective effect of ACL in zebrafish, we conducted experiments on both wildtype and *hmox1a*^−/−^ larvae. First, we examined the mRNA level of *hmox1a* in wildtype and *hmox1a*^−/−^ larvae treated with FAC and ACL. Remarkably, *hmox1a*^−/−^ larvae exhibited lower *hmox1a* mRNA levels (decreased 95.1%), regardless of the presence of ACL and FAC, thus validating the efficacy of this transgene line (Figure 7A). Next, we measured ROS production in embryos from both zebrafish lines to determine if the *hmox1a* knockout influenced ROS biogenesis. ACL exposure led to decreased ROS production in wildtype larvae (decreased 2.23-fold), highlighting its protective effect. Notably, the protective effect of ACL was diminished in *hmox1a*^−/−^ larvae (Figure 7B,C and Figure S5). To further confirm the impact of the *hmox1a* gene on ferroptosis, we determined the mRNA expression level of ferroptosis-related genes in zebrafish larvae and *hmox1a*^−/−^ larvae treated with FAC with or without ACL. ACL treatment resulted in downregulated expression of ferroptosis-related genes (*slc34a2a* and *loc558816*) in wildtype larvae. However, this effect was significantly weakened in *hmox1a*^−/−^ larvae (Figure 7D,E). Moreover, we determined osteogenesis-related indicators on zebrafish larvae (7 dpf) to assess the impact of the *hmox1a* gene on the osteogenic effect of ACL. We observed an increase in vertebral fluorescence intensity and vertebrae density in ACL treated wildtype larvae, indicative of enhanced bone mineralization. Surprisingly, *hmox1a*^−/−^ larvae treated with ACL did not exhibit the same increase in vertebral fluorescence intensity (Figure 7F,G and Figure S6). Furthermore, we analyzed the mRNA expression levels of osteogenesis-related genes, *spp1*, *bglap*, and *runx2a*, in wildtype and *hmox1a*^−/−^ zebrafish larvae. The results demonstrated that ACL treatment upregulated the expression of these genes in wildtype larvae. However, this effect was notably attenuated in *hmox1a*^−/−^ larvae (Figure 7H–J).

## 4. Discussion

Iron overload-induced osteoporosis presents a significant challenge in bone metabolism research. Understanding the molecular mechanisms behind this condition is crucial for developing effective therapeutic strategies. This study examines the effects of ACL on bone metabolism under FAC-induced iron overload. The study examined the mechanisms that underlie the protective effects of ACL against iron overload-induced inhibition of osteogenesis. Zebrafish larvae and MC3T3-E1 osteoblast cells were used as in vivo and in vitro models. The results indicate that ACL mitigates the inhibition of bone formation caused by iron overload through HO-1.

Consistent with previous research on ACL toxicity [16,17,18,19], we observed that ACL exposure above 1 mg/L led to developmental defects and decreased survival in zebrafish embryos, indicating a concentration-dependent biological effect. These findings are consistent with previous studies reporting adverse health effects of areca nut chewing, including neurological impairment [19]. These findings guided our selection of appropriate ACL concentrations for further experiments. Remarkably, despite its toxicity at high doses, low-dose ACL alleviated the inhibition of mineralization caused by FAC exposure in zebrafish larvae, as evidenced by enhanced alizarin red staining and upregulated expression of osteogenic markers like *spp1*, *bglap*, and *runx2a* (Figure 2). The study validated the osteoprotective effect of ACL in MC3T3-E1 osteoblasts. ACL attenuated the suppression of mineralization, alkaline phosphatase activity, and osteogenic gene expression induced by FAC treatment (Figure 3). These observations suggest that ACL has the potential to modulate both osteoblast and osteoclast activity, consistent with a previous study reporting the inhibitory effects of ACL on RANKL-induced osteoclast differentiation in vitro [7]. However, further research is needed to determine the specific effects of ACL on osteoclasts under iron overload.

Through RNA sequencing analysis, we found evidence that the ACL may regulate ferroptosis pathways in order to exert its protective effects on osteogenesis (Figure 4). This hypothesis led us to systematically evaluate features of ferroptosis in downstream experiments. As described above, ACL suppressed ferroptosis by ameliorating iron-induced lipid peroxidation, mitochondrial dysfunction, ROS generation and cell death. RNA-seq analysis identified potential candidate genes involved in the modulation of ferroptosis and osteogenesis by ACL. The study focused on genes related to ferroptosis, and discovered that ACL specifically upregulated *hmox1a*, which encodes heme oxygenase-1 (HO-1). HO-1 is a master regulator of iron recycling and oxidative stress [20] (Figure 4C). These sequence results guided subsequent functional studies, establishing HO-1 as a critical mediator of ACL to inhibit ferroptosis and promote osteoblast differentiation.

Ferroptosis, which is characterized by the accumulation of iron, lipid peroxidation, and oxidative damage, has recently been identified as a contributing factor to osteoporosis [21,22]. Our study provides a novel mechanism for the modulation of bone metabolism, and is the first to link ACL to the regulation of ferroptosis. Specifically, we identified heme oxygenase-1 (HO-1) as a key mediator of ACL’s inhibition of ferroptosis and promotion of osteogenesis under FAC treatment. HO-1 catalyzes the degradation of heme into biliverdin, free iron, and carbon monoxide [23], serving as a sensor and regulator of intracellular iron levels and oxidative stress [24]. HO-1 has been shown to be protective against oxidative stress and cell damage induced by iron overload through heme degradation [25]. ACL treatment significantly upregulated HO-1 expression in both zebrafish larvae and MC3T3-E1 cells under FAC exposure. Through HO-1 knockdown experiments, we demonstrated that HO-1 is essential for ACL’s downregulation of ferroptosis markers and upregulation of osteogenic genes (Figure 6). Furthermore, knockout of the zebrafish ortholog, *hmox1a*, attenuated ACL’s inhibition of ferroptosis and its enhancement of mineralization (Figure 7). Partial inhibition of HO-1 attenuated the osteogenic-promoting effects of ACL, suggesting the involvement of HO-1 in the regulation of ferroptosis and osteogenesis of ACL. These findings are consistent with previous studies that emphasize the critical role of HO-1 in ferroptosis regulation [8,9].

The relationship between HO-1 and ferroptosis is complex and dynamic. HO-1 plays an important role in cellular defense mechanisms against oxidative stress and lipid peroxidation. By regulating iron homeostasis and alleviating oxidative stress, HO-1 activation can inhibit ferroptosis [26]. HO-1 breaks down heme, releasing iron, biliverdin and carbon monoxide. This reduces intracellular iron levels, which suppresses iron-mediated lipid peroxidation, a core process in the execution of ferroptosis [27]. Additionally, the metabolites of heme degradation, such as bilirubin, have antioxidant effects that mitigate oxidative damage involved in ferroptosis. However, some studies have suggested that sustained overactivation of HO-1 may also promote ferroptosis and tissue injury. Excess HO-1 activity can lead to iron overload by releasing high levels of iron from heme, thus creating a pro-ferroptotic environment [28]. The free iron can participate in Fenton reactions, generating reactive oxygen species and propagating lipid peroxidation. Furthermore, high CO levels from HO-1 overexpression may impair mitochondria, sensitizing cells to oxidative damage [29]. Therefore, HO-1 acts as a double-edged sword—while moderate induction of HO-1 is protective, chronic HO-1 hyperactivity can be detrimental and pro-ferroptotic. The effects of HO-1 on ferroptosis appear to be context-dependent based on factors like cell type, stimuli, and disease state [30]. Maintaining HO-1 homeostasis is crucial, as both inadequate and excessive HO-1 can potentially facilitate ferroptosis-mediated cell death and tissue injury. Elucidation of the role of HO-1 in the regulation of ferroptosis has broad implications for the development of novel therapeutic strategies against diseases characterized by ferroptosis-induced cell death [28].

Zebrafish (*Danio rerio*) have emerged as a valuable vertebrate model for studying development, diseases, and drug effects, due to advantages including optical transparency, rapid development, high fecundity, and conserved genetics with mammals [31]. Our work adds to existing literature highlighting the potential of zebrafish larvae for screening compounds that modulate bone formation [32,33]. By taking advantage of the optical transparency of zebrafish larvae, we assessed the impact of ACL on oxidative stress and iron metabolism by quantifying ROS levels and ferroptosis biomarkers (Figure 5). Overall, zebrafish enabled thorough in vivo evaluation of ACL’s osteogenic and anti-ferroptotic effects. Using CRISPR/Cas9 technology, we successfully developed a *hmox1a* knockout zebrafish line. Very few studies have examined the biological role of zebrafish *Hmox1a* to date [34]. In a study using an *hmox1a* knockout zebrafish line, the authors found that zebrafish *Hmox1a* counters infection by limiting iron accumulation and ferrostatin-sensitive cell death [34].

Some limitations should be noted regarding our experimental design. First, we used a prophylactic protocol by applying ACL concurrently with FAC, rather than studying its effects after pre-established iron overload. It would better mimic clinical scenarios to test a therapeutic regimen. Second, we studied only osteoblast responses. Including osteoclast models would provide a more holistic perspective. Finally, additional testing is needed to optimize dosage and safety of ACL before translating these findings into clinical applications.

## 5. Conclusions

**Figure 8:**
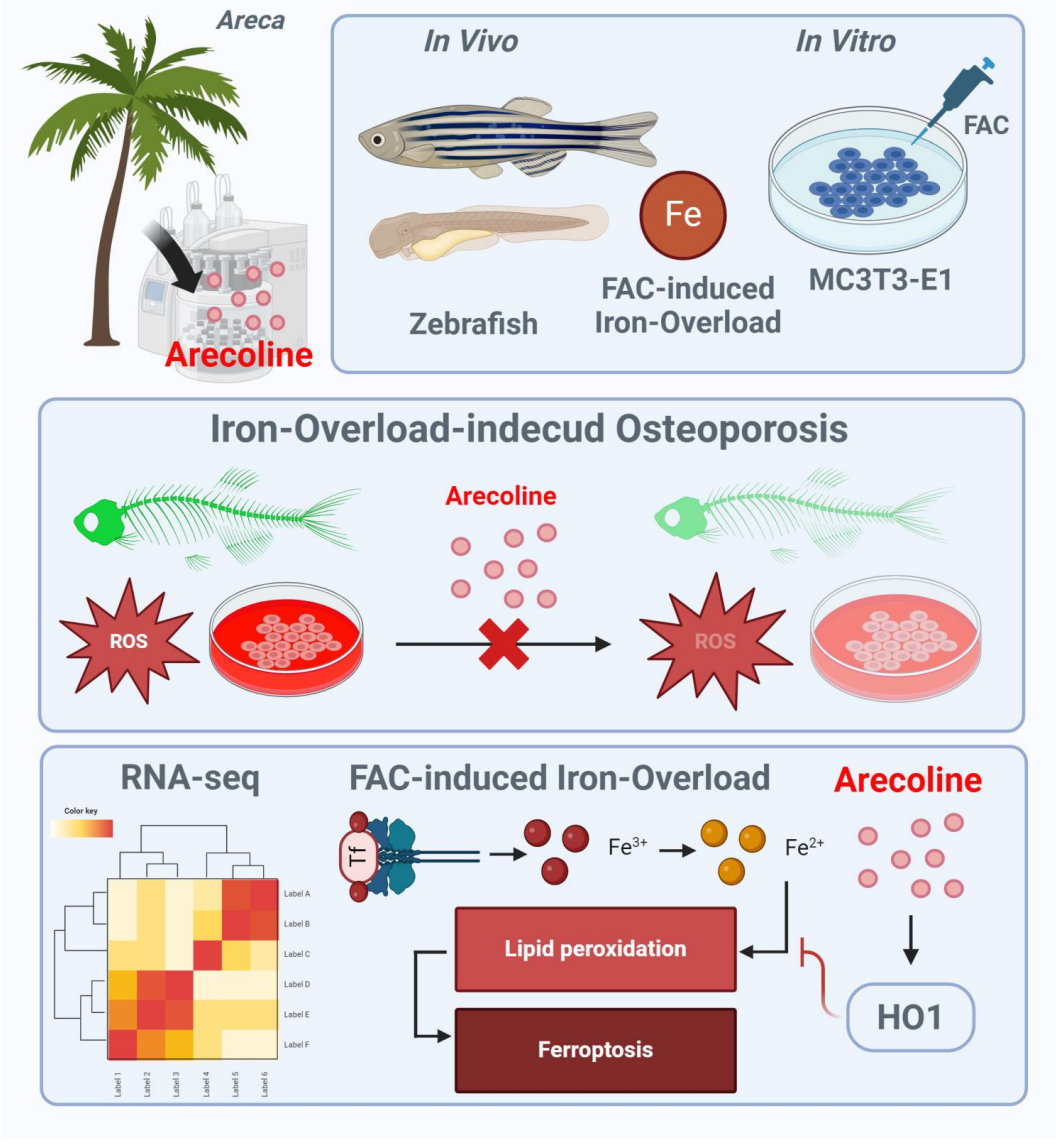
Schematic illustration of ACL for protecting FAC induced osteoporosis via upregulation of HO-1. FAC treatment leads to increased intracellular iron levels, lipid peroxidation, mitochondrial dysfunction, and oxidative stress, contributing to the inhibition of osteoblast differentiation and progression of osteoporosis. ACL attenuates these inhibitory effects by upregulating HO-1 expression. HO-1 activation promotes iron recycling and exerts antioxidant effects, thereby suppressing hallmarks of ferroptosis. The illustration figure was created by BioRender.

This study shows that ACL improves osteogenesis and inhibits ferroptosis in the presence of iron overload. The activation of HO-1 is identified as a key mechanism underlying these effects. The findings expand the current understanding of ACL beyond its risks and explore its therapeutic potential in bone metabolism (Figure 8).

## Data Availability

Data are contained within the article and Supplementary Materials.

## Supplementary Materials

The following supporting information can be downloaded at: https://www.mdpi.com/article/10.3390/antiox13040430/s1.
