# Supplementary material for: A Mechanistic Study of the Osteogenic Effect of Arecoline in an Osteoporosis Model: Inhibition of Iron Overload-Induced Osteogenesis by Promoting Heme Oxygenase-1 Expression
Source: Antioxidants (Basel). 2024 Mar 31;13(4):430. doi: 10.3390/antiox13040430 (PMC11047558; doi:10.3390/antiox13040430)
Supplement: Supplementary file 1 [file antioxidants-13-00430-s001.zip › Supplementary figures ACL.pdf]

Supplementary figures

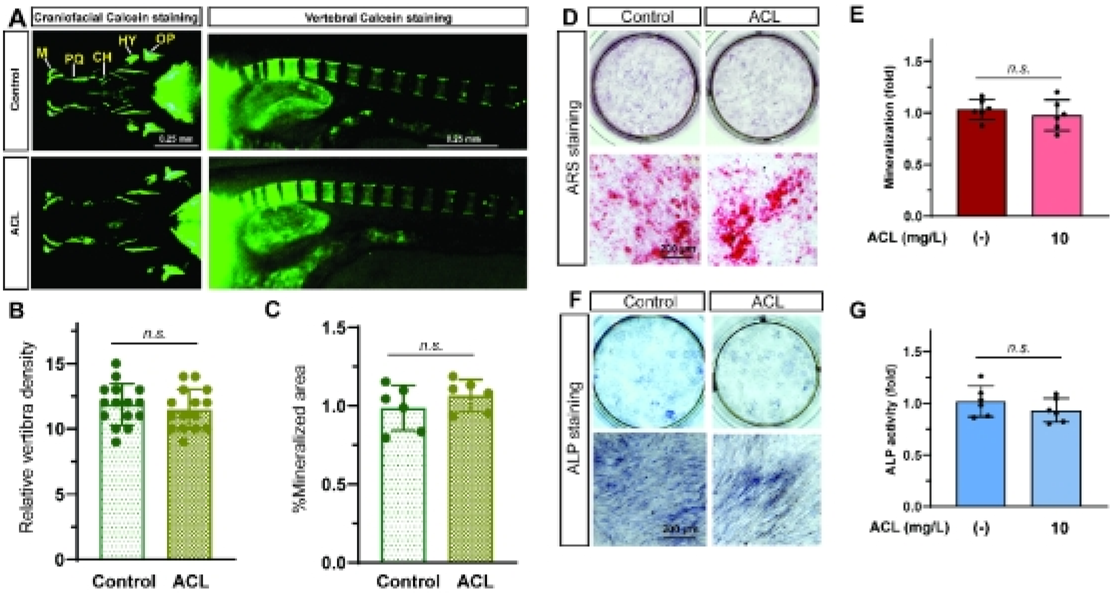

Fig S1. The effects of FAC on the bone formation of zebrafish larvae and the osteogenic differentiation of MC3T3-E1 cells under FAC treatment.

(A) Calcein staining of zebrafish larvae (7 dpf) cranium from control or 0.5mg/L ACL treatment groups. (B) Mineralized areas were quantified and showed in the graph. (C) The number of calcein-stained vertebral bodies were calculated. (D-G) Alizarin red staining and Alkaline phosphatase staining of MC3T3-E1 cells treated 10 mg/L ACL for 1 or 2 weeks. Degree of mineralization and Alkaline phosphatase activity were quantified, * means $P < 0.05$ between two indicated groups (n=6).

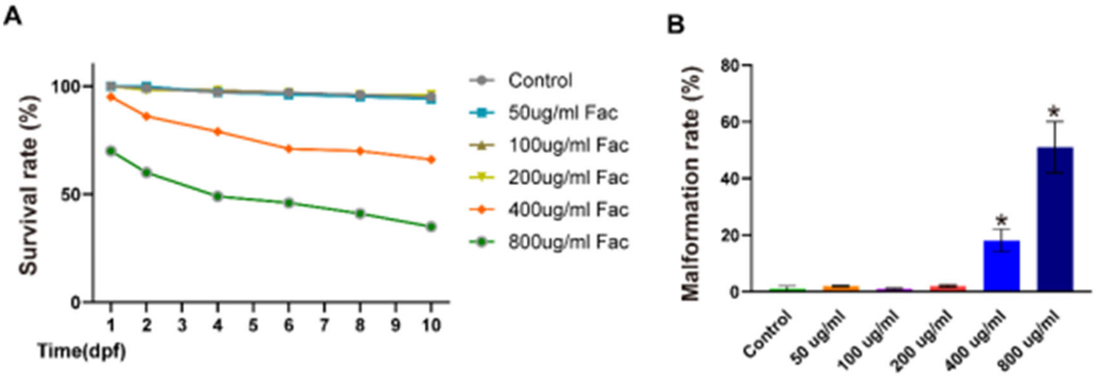

Fig S2. The effects of different concentrations of FAC on the survival rate and malformation rate of zebrafish larvae.

(A) Survival rate of zebrafish larvae at different concentrations of FAC within 10 dpf. (B) Malformation rate of zebrafish larvae at different concentrations of FAC for 10 days. n=50.

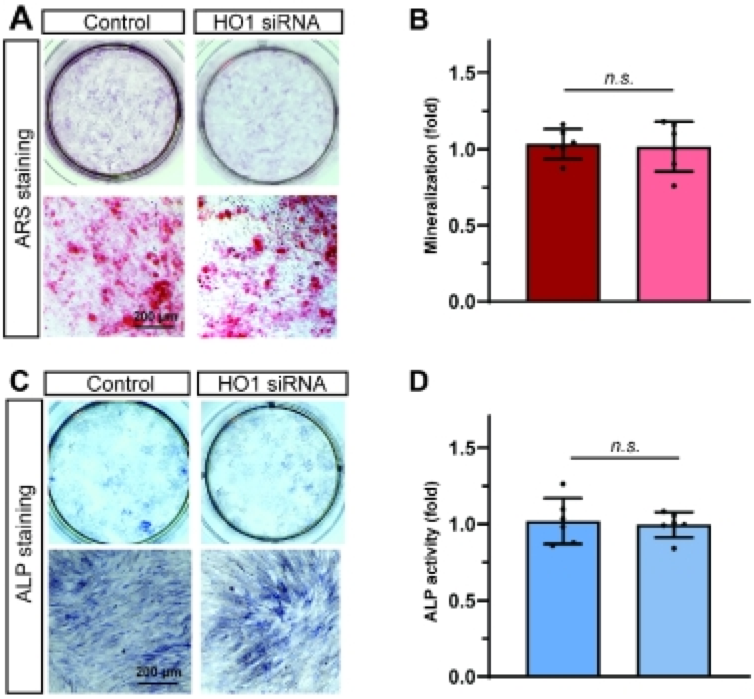

Fig S3. HO-1 knockdonw has no effect on the osteogenic differentiation of MC3T3-E1 cells.

(A) Alizarin red staining of MC3T3-E1 cells transfected with HO1-siRNA for 2 weeks. (B) Degree of mineralization were quantified, * means $P < 0.05$ between two indicated groups (n=6). (C) Alkaline phosphatase staining of MC3T3-E1 cells transfected with HO1-siRNA for 1 week. (D) Degree of Alkaline phosphatase activity were quantified, * means $P < 0.05$ between two indicated groups (n=6).

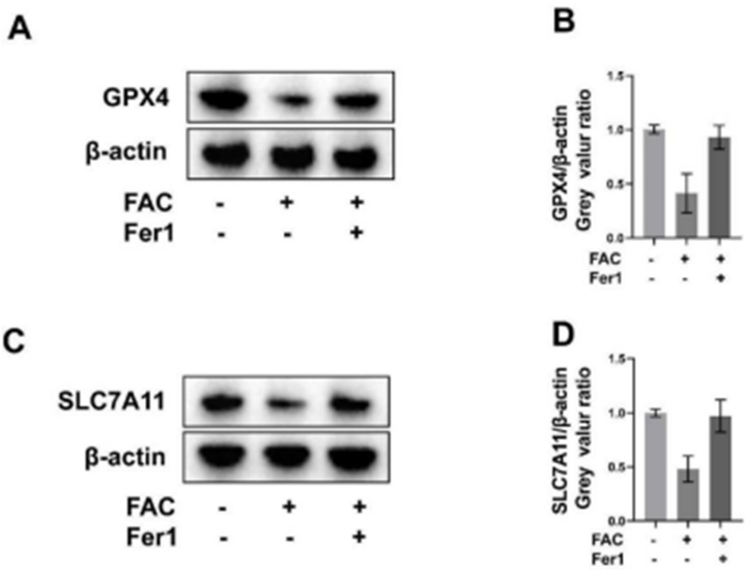

Fig S4. The effect Fer1 on ferroptosis induced by FAC.

(A-D) The expression of ferroptosis associated genes, GPX4 and SLC7A11, in control, FAC, FAC + Fer1 treated MC3T3-E1 cells were determined by Western blotting. β-actin was used as internal reference. Relative expression levels were shown in the graph. * means $P < 0.05$ between two indicated groups (n=3). All data are displayed as mean ± SD.

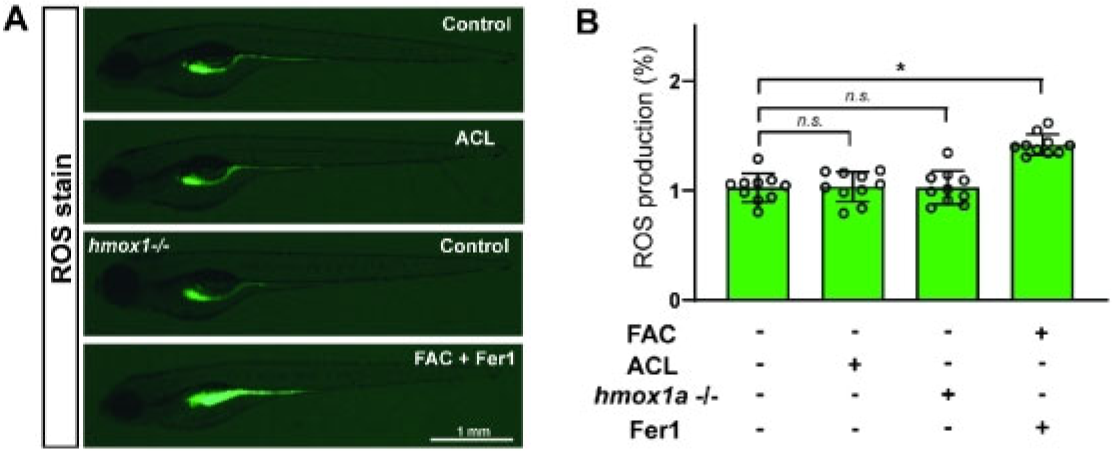

Fig S5. The effect of HO-1 knockout, ACL and FAC + Fer1 on ROS production of zebrafish larvae. (A, B) ROS production of embryos (5dpf) from two zebrafish lines, wildtype and *hmox1a* -/- larvae treated with ACL and FAC + Fer1 were detected and quantified by staining with DCF-DA, * means $P < 0.05$ between two indicated groups (n=10).

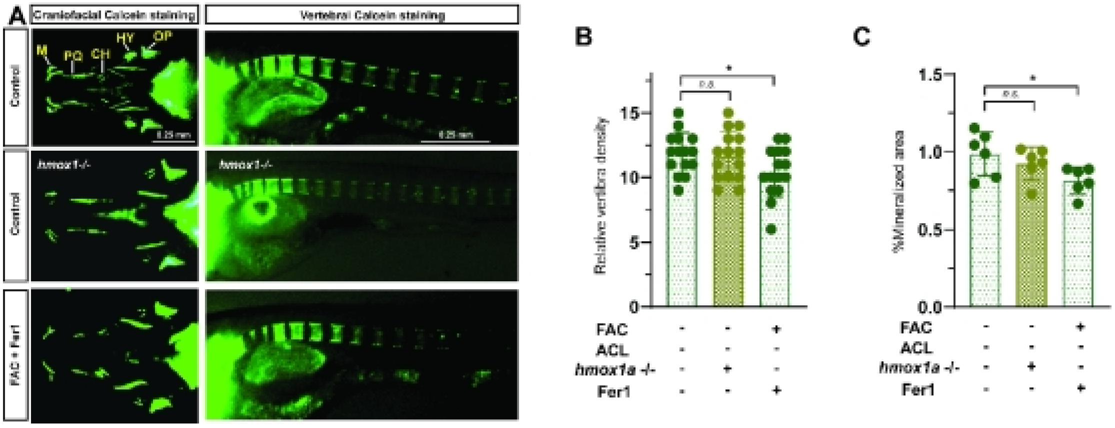

Fig S6. The effect of HO-1 knockout, ACL and FAC + Fer1 on ROS production of zebrafish larvae. (A, B) ROS production of embryos (5dpf) from two zebrafish lines, wildtype and *hmox1a* -/- larvae treated with ACL and FAC + Fer1 were detected and quantified by staining with DCF-DA, * means $P < 0.05$ between two indicated groups (n=10).
